# Supplementary material for: A single-cell transcriptomic atlas reveals the emergence of medusa-specific cell states in the scyphozoan Aurelia coerulea
Source: PLoS Biol. 2026 Aug 14;24(8):e3003931. doi: 10.1371/journal.pbio.3003931 (PMC13485128; doi:10.1371/journal.pbio.3003931)
Supplement: Supplementary file 1 [file pbio.3003931.s001.html]

Aurelia Atlas | Supplementary Data Figures


# Aurelia Atlas | Supplementary Data Figures

**Aurelia Cell Atlas:**

Supplementary materials in support of the conclusions drawn in Link
et al. (2026), PLOS Biology.

**A single-cell transcriptomic atlas reveals the emergence of
medusa-specific cell states in the scyphozoan *Aurelia coerulea***    
  
 Oliver Link1, Stefan M. Jahnel2, Kristin Janicek1,
Daniel Guerguerian1, Johanna Kraus1, Juan Daniel Montenegro1, Bob
Zimmerman1, Brittney Wick3, Konstantin Khalturin4, Alison G. Cole1\*, and
Ulrich Technau1,5

### **Figure A: Life cycle: medusa genes**

Fig A: **a**) Total number of genes (nFeatures\_RNA) and
**b**) molecules (nCounts\_RNA) recovered from all cells
captured across life cycle stages **c**) Distribution of
medusa-specific genes across all cell types. The set of all
medusa-specific genes were considered. For every cluster the gene is
counted as expressed if reads are detected and expression value is above
the average expression across all identities.

### **Figure B: Life cycle: differentially expressed genes**

**Fig B: Differential gene usage across lifecycle. a**)
Dotplot of the set of deferentially expressed genes across life cycle
stages. Plot split in two panels for increased legibility.
**b:e**) TOP go evaluation of all differentially expressed
genes across life cycle stages. See supplementary Data S1.2 for full
gene lists.

### **Figure C: Full cell state complement**

**Fig C: Full cell state complement**. UMAP
representation of all cluster identities, separated by life cycle
stage.

### **Figure D: Digestive Gland**

**Fig D: Digestive gland population**.
**a**) Full dataset UMAP showing position of cluster subset
**c**) UMAP distribution of identified clusters.
**c**) Bar plot showing distribution of digestive gland
cells across life cycle stages and medusa tissue samples as absolute
cell numbers. **d**) Gene expression dot plot showing
expression profile of *ashC* specifically in identified digestive
gland cell subtypes. **e**) Gene expression dot plot
showing expression profile of top 10 differentially expressed genes for
each cluster, separated by life cycle contribution. Full gene list is
available in supplementary data S1.10 **f)** *in
situ* hybridization showing localization of chitinase gene
expression to the gastric filaments.

### **Figure E: Mucin gland cluster**

**Fig E: Mucin gland population**. **a**)
Full dataset UMAP showing position of cluster subset **b**)
UMAP distribution of identified clusters. **c**) Bar plot
showing distribution of mucin gland cells across life cycle stages and
medusa tissue samples as absolute cell numbers. **d**) Gene
expression dot plot showing expression profile of mucin-like-protein
(*mlp-like*) and the bHLH transcription factor *ngn*
specifically in identified mucin gland cell subtypes. One paralog of the
*atoh8* gene family is also expressed specifically in mucin
subtypes *mucin.3* and *mucin.medusa*.

## **Figure F: Myc gene tree**

**Fig F: Cnidarian expansion of myc-family members. a**)
Maximum likelihood tree; *Aurelia* is indicated in blue,
*Nematostella* in salmon, other cnidarians in black and
bilaterian orthologs in grey. Expression within the two shared cell type
states (early and mature) as indicated in Figure 3 are plotted. Internal
nodes indicate bootstrap values. Ac: *Aurelia coerulea*; Bf:
*Branchiostoma floridae*; Ce: *Caenorhabditis elegans*;
Cx: *Cassiopea xamachana*; Ed: *Exaiptasia diaphana*; Hs:
*Homo sapiens*; Hv: *Hydra vulgaris*; Hy: *Hydractinia
symbiolongicarpus*; Mg: *Magallana gigas*; Nv:
*Nematostella vectensis*; Of: *Owenia fusiformis*; Re:
*Rhopilema esculentum*; Sm: *Sanderia malayensis*; Sp:
*Stylophora pistillata* ; Tc: *Tripedalia cystophora*
**b**) Expression of included gene models on the *Hydra
vulgaris* dataset (from *https://research.nhgri.nih.gov/HydraAEP/SingleCellBrowser/*).

### **Figure G: Neuron GO terms**

**Fig G: Over-represented biological processes in differential
gene lists (topGO)**. Each set of up-regulated genes from all
neuroglandular subtypes was evaluated for over-represented biological
processes with the topGO package. Full gene lists are available in
supplementary data S1.8.

### **Figure H: ATOH8 family genes**

**Fig H: Expression of atoh8 family transcription
factors.** **a**) Expression profiles across all
identified cell type families of each member of the atoh8 family.
**b:d**) Expression profiles of atoh8 family genes within
the neural clusters of *Aurelia* (b), *Nematostella* (c),
and *Hydra* (d) highlights both broad expression profiles, as
well as cell type specific expression. Expression of included gene
models on the *Hydra vulgaris* dataset (from *https://research.nhgri.nih.gov/HydraAEP/SingleCellBrowser/*).
G023106 is the hydra *pou4* ortholog indicating class n2-type
neurons, and G017257 is the *ins* ortholog, indicating class
n1-type neurons. **e**) Gene family tree with
*Nematostella* (salmon), *Aurelia* (blue), *Hydra*
(blue-green) and human (red) paralogs highlighted. Internal nodes
indicate bootstrap values. Ac: *Aurelia coerulea*; Bf:
*Branchiostoma floridae*; Ce: *Caenorhabditis elegans*;
Cx: *Cassiopea xamachana*; Ed: *Exaiptasia diaphana*; Hs:
*Homo sapiens*; Hv: *Hydra vulgaris*; Hy: *Hydractinia
symbiolongicarpus*; Mg: *Magallana gigas*; Nv:
*Nematostella vectensis*; Of: *Owenia fusiformis*; Re:
*Rhopilema esculentum*; Sm: *Sanderia malayensis*; Sp:
*Stylophora pistillata* ; Tc: *Tripedalia cystophora*

### **Figure I: Muscle Transcription Factors**

**Fig I:** **Muscle-specific transcription
factors.** Dotplot of expression of the top differentially
expressed gene models with DNA binding sites specific to the striated
muscle subset. Full gene list is available in supplementary Data
S1.4b.
